# Study on the Relation Between Polished Surface Integrity and Fatigue Behavior of Low-Alloy Steel

**DOI:** 10.3390/ma19071284

**Published:** 2026-03-24

**Authors:** Yong Wang, Yang Xiao, Dongfei Wang, Xibin Wang, Zhibing Liu, Kun Xu

**Affiliations:** 1 College of Mechanical and Vehicle Engineering, Taiyuan University of Technology, Taiyuan 030024, China; 2 Key Laboratory of Fundamental Science for Advanced Machining, Beijing Institute of Technology, Beijing 100081, China; 3 Jinxi Axle Company Limited, Taiyuan 030024, China; 4 Zhengzhou Research Institute of Mechanical Engineering Co., Ltd., Zhengzhou 477150, China

**Keywords:** surface roughness, residual stress, micro pitting fatigue, gear steel

## Abstract

The fatigue pitting model based on the minimum oil film thickness does not consider the influence of tooth surface roughness and residual stress, which limits the accuracy of predicting the fatigue pitting of the model. Micro pitting often initiates on the surface due to large external loads. Therefore, it is urgent to propose a new-micro pitting bearing capacity model based on gear surface integrity parameters. This paper studied a new fatigue pitting model considering surface integrity subjected to polishing processes. This model thoroughly analyzes the effects of teeth surface roughness dynamically on the oil film pressure and explores the complex mechanism of residual stress in the near-surface stress field of the gear teeth. The new model can more accurately simulate the micro-pitting bearing capacity under actual operating conditions by introducing teeth surface roughness and residual stress, and the prediction reliability of gear steel is greatly improved. This improved model provides a solid theoretical basis and technical support for optimizing gear transmission systems, accurate diagnosis of micro-pitting defects, and in-depth theoretical research in related fields.

## 1. Introduction

In modern mechanical transmission systems research and application, gears are core components. Their performance stability and reliability directly influence the entire system’s operational efficiency and service life. Micro pitting, a prevalent and severely damaging form of gear failure, has long been a focal point in academic and engineering domains. As emphasized by Mallipeddi et al. [1], the occurrence of micro pitting not only reduces the transmission efficiency of gears but may also trigger a chain reaction. Moreover, it leads to malfunctions in the entire mechanical system and severely affects the regular operation of equipment and production efficiency. Therefore, detailed research into micro-pitting and accurate assessment of its load-bearing capacity is essential. This is essential to ensure the mechanical transmission systems’ stable operation and extend their service life.

Numerous studies have indicated that teeth surface roughness and residual stress are pivotal factors in determining the micro-pitting bearing capacity. The roughness on the teeth’ surface can break the continuity of the oil film. As a result, it leads to sharp fluctuations in the oil-film pressure in some local regions. This, in turn, significantly impacts the initiation and development of micro-pitting. Everitt [2] investigated the influence mechanism of teeth surface roughness on gear contact stress distribution. Their findings indicated that teeth surface roughness can lead to non-uniform stress distribution, which is closely related to the initiation of micro-pitting. This provides additional evidence for the importance of teeth surface roughness in micro-pitting research. Bergstedt [3] performed micro-pitting and pitting tests on standard FZG PT–C and GF–C gears on an FZG back-to-back test rig. They accurately measured the changes in the gear teeth profile after the tests due to micro-pitting and pitting damage and calculated the gear surface roughness parameters in detail. The research findings demonstrate that when the teeth surface roughness increases, it undermines the continuity of the oil film. This leads to significant fluctuations in the local oil-film pressure. Consequently, the initiation and expansion of micro-pitting are accelerated. The physics-based model proposed by Li and Kahraman [4] profoundly expounds on the influence of surface roughness topography on local stress concentration, further emphasizing the key role of teeth surface roughness in the occurrence process of micro-pitting. In addition, Vrček et al. [5] revealed, through microscopic observation and simulation analysis, how the oil-film pressure fluctuations induced by teeth surface roughness led to changes in asperity contact stress and thus affected the initiation position and development direction of micro-pitting.

Residual stress also profoundly impacts the teeth surface stress field, significantly influencing the micro-pitting load-carrying capacity. Residual tensile stress raises the actual stress level on the teeth surface and lowers the threshold for the initiation of micro-pitting. On the contrary, residual compressive stress can impede the occurrence of micro-pitting.

Based on Continuum Damage Mechanics (CDM), He et al. [6] concluded that the initial compressive stress positively affects contact fatigue performance. In contrast, the initial tensile stress significantly reduces the contact fatigue life of gears. Wang et al. [7] thoroughly explored the influence of the residual stress state on the load-carrying capacity of the case-hardened gears, clarifying the crucial role of residual stress in the micro-pitting process. In addition, Liu et al. [8] found that residual compressive stress can effectively delay the appearance of micro-pitting, while residual tensile stress accelerates the development of micro-pitting. Liu et al. [9] combined the quantitative relationship between residual stress and micro-pitting damage with finite-element analysis. Li [10] applied the Dang Van criterion to predict micro-pitting failure in gear transmission systems. Their research results can be used as a reference for the application of this criterion in the micro-pitting, load-carrying capacity model of this study.

Nevertheless, most traditional micro-pitting, load-carrying capacity models are built upon the minimum oil-film thickness [11,12,13]. As a result, they cannot comprehensively consider crucial factors like tooth surface roughness and residual stress. This limitation severely restricts the accuracy of predicting the micro-pitting load-carrying capacity. Morales-Espejel [14] also pointed out that traditional models neglecting surface topography and lubrication conditions may lead to inaccurate predictions of micro-pitting load-carrying capacity, consistent with the limitations discussed in this study. Actual cases enumerated by Zhang et al. [15] show that some gear transmission systems designed according to traditional models still frequently experience micro-pitting failures during actual operation, and it causes considerable losses to production. In addition, by comparing the prediction results of traditional models with the actual occurrence of micro-pitting, Brandão et al. [16] found that due to the neglect of teeth surface roughness and residual stress, the error between the predicted micro-pitting load-carrying capacity and the actual value can reach 20–30%, seriously affecting the design reliability of the gear transmission system.

**Figure 1:**
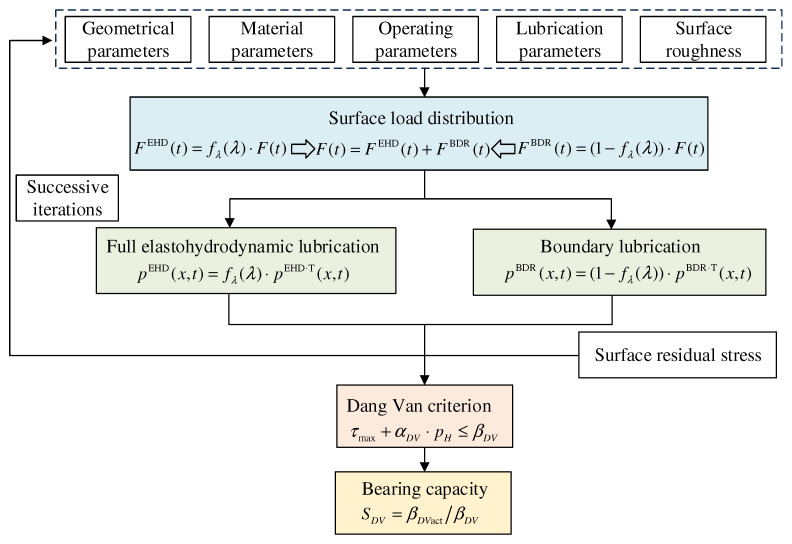
A micro-pitting bearing capacity model considering teeth surface roughness and residual stress.

Considering the above background and current state of affairs, this research innovatively develops an enhanced micro-pitting, load-carrying capacity model. This model comprehensively incorporates two critical factors: teeth surface roughness and residual stress. During the model-construction phase, in-depth exploration is carried out on how teeth surface roughness dynamically impacts the oil-film pressure and the intricate mechanism of how residual stress functions within the teeth surface stress field. By integrating these two key factors into the model, the model can more accurately simulate the gears’ micro-pitting load-carrying capacity under real-world operating conditions. Consequently, the reliability of the prediction results is significantly enhanced. This improved model is anticipated to be pivotal in practical engineering applications. It can substantially boost the reliability and stability of gear transmission systems, offering robust theoretical support and technical assurance for practical engineering applications. As shown in Figure 1 of the micro-pitting bearing capacity model, model construction can be divided into two core stages:

## 2. Stress Distribution Analysis of Teeth Surface

### 2.1. Surface Roughness and Residual Stress Measurements

**Figure 2:**
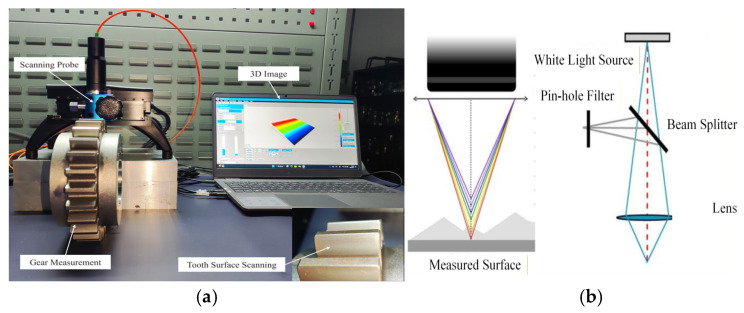
Appearance and working principle of the 3D surface profilometer. (**a**) Composition of the 3D surface profilometer and (**b**) measurement principle.

The surface roughness of the gear specimens was measured using a three-dimensional profilometer (Nanovea, Irvine, CA, USA). As shown in Figure 2, the scanning area was 2 mm × 2 mm, with a 1 μm step, allowing high-precision reconstruction of the gear-tooth surface morphology. The instrument operates based on optical dispersion and confocal principles, achieving a minimum step of 0.1 μm and a maximum measurable area of 50 mm × 50 mm. These settings bound the topography-measurement resolution; the 1 μm scan step defines the grid-sampling discretization, while the 0.1 μm minimum step provides the lower limit for vertical resolution and thus the instrument-limited uncertainty of roughness metrics reported in this study.

The residual stress of the test gears was measured using a high-power X-ray stress analyzer (Stresstech Oy, Jyväskylä, Finland). The measurement instrument, specific test-point locations, and depth information are illustrated in detail. Measurements were taken at the critical meshing points of the tooth-root region, with a 0.1 mm interval and a maximum depth of 2.0 mm, to reveal the longitudinal distribution of residual stress inside the gear. The 0.1 mm depth increment defines the depth-profiling resolution and the associated discretization uncertainty of the X-ray residual-stress profile up to 2.0 mm; interpretation of near-surface gradients is therefore constrained by this increment.

### 2.2. Gear Specimens and Materials

**Figure 3:**
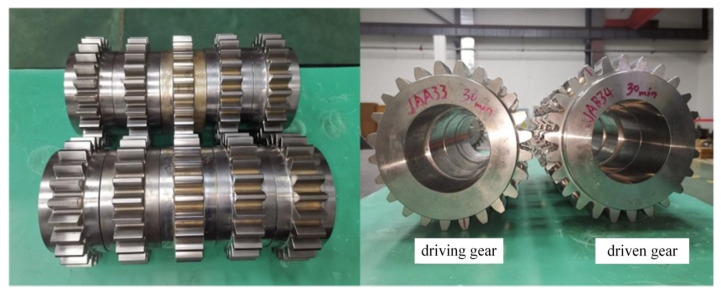
The test gear specimen.

In this study, the test specimens were standard spur involute gear pairs, and their geometrical parameters are listed in Table 1. Partial views of the driving and driven gear specimens are shown in Figure 3.

The test gears were made of low-carbon alloy steel 18CrNiMo7-6, and the chemical composition is given in Table 2.

After carburizing and quenching heat treatment, this material exhibits exceptionally high tensile strength and excellent wear resistance. The low carbon content helps maintain good toughness and ductility, preventing brittle fractures in regions with high stress concentration. In addition, alloying elements such as chromium (Cr), nickel (Ni), and molybdenum (Mo) strengthen the matrix structure, increasing both tensile and yield strength, while also enhancing corrosion resistance and thermal stability. Moreover, the hard carbides formed during carburizing significantly improve the surface hardness of the gear and thus its resistance to wear.

In practical applications, after carburizing and quenching, the surface layer of 18CrNiMo7-6 steel forms a moderately deep and highly hardened case, while the core retains adequate toughness. This ideal combination of a hard surface and tough core ensures that the gear can withstand high contact stresses while maintaining excellent resistance to fatigue crack initiation and propagation.

In this experiment, the lubricating oil used was the Shell L-CKD series industrial gear oil, and its main physical properties are listed in Table 2 and Table 3. To ensure the stability and accuracy of the testing conditions, the inlet oil temperature was controlled at 60 °C, and the oil flow rate was maintained at 22 L/min.

### 2.3. Gear Contact Fatigue Test Rig

The experimental setup used in this study is a back-to-back high-performance gear contact fatigue test rig. The test rig consists of a driving system, loading system, gearbox assembly, lubrication system, and monitoring and control system. The main driving source of the test rig is an electric motor, which transmits rotational power to the driving gear through a belt pulley transmission system. The loading system applies a constant torque to the driven gear via a hydraulic actuator.

**Figure 4:**
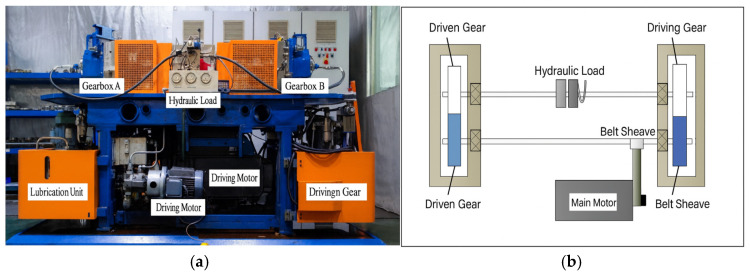
Gear contact fatigue test rig and its schematic diagram. (**a**) Photograph of the test rig. (**b**) Schematic diagram of the test rig.

The test rig is equipped with two completely independent gearboxes (Box A and Box B). The gear pairs inside the two gearboxes are connected back-to-back via an elastic shaft, an isolation design that effectively reduces vibration interference between the gear pairs, ensuring that each test operates independently and thereby improving the accuracy of the results. Each gearbox is equipped with an independent lubrication supply system, enabling real-time control of oil quantity and temperature. The complete physical structure of the test rig is shown in Figure 4a. The loading unit adopts an intelligent control system integrating both electrical and hydraulic circuits, and its operating principle is illustrated in Figure 4b. The main motor transmits rotational speed to the driving gear via the belt system.

**Figure 5:**
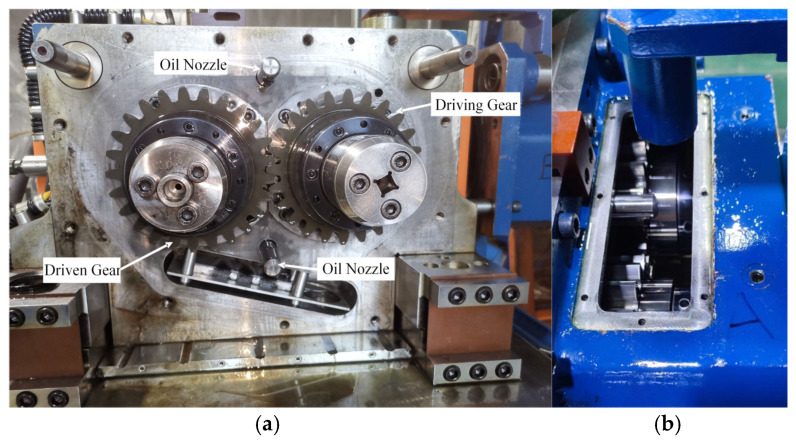
Gearbox structure of the fatigue test rig. (**a**) Internal structure of the gearbox. (**b**) Observation window.

The main motor of the test rig has a rated power of 75 kW, and the maximum circulating power between the two gearboxes reaches 2000 kW. The maximum rotational speed is 4500 r/min, and the maximum torque is 6000 Nm. The gearboxes employ a spray lubrication method, which allows the precise control of oil injection during gear operation to form a stable oil film, effectively reducing friction and wear between gear surfaces. Each gearbox is equipped with two oil nozzles, as shown in Figure 5a. To facilitate the observation of changes in the gear surface morphology, observation windows are installed on the upper part of each gearbox, as illustrated in Figure 5a,b.

The fatigue test rig integrates a comprehensive and precise real-time monitoring and control system, ensuring full control of all key performance parameters throughout the testing process. This advanced control system provides dynamic adjustment and real-time monitoring of rotational speed, torque, as well as lubricant inlet temperature and flow rate, maintaining all testing conditions within the designated optimal range to ensure the accuracy and reliability of the results. In addition, the system can record and store critical data in real time, including but not limited to the actual power output, vibration parameters of the gearbox, performance metrics of the lubrication system, and the cumulative number of load cycles during the fatigue testing process.

### 2.4. Model Assumptions and Boundary Conditions

However, to simplify the numerical solution process of the elastohydrodynamic lubrication (EHL) model and improve the analysis efficiency [18,19], the actual contact problem of gears is often approximated as a two-dimensional line-contact model. This treatment transforms the complex surface contact into the rolling contact between two equivalent cylinders, which is a simplified representation derived from the Hertzian elliptical contact theory. Such an approximation greatly reduces the computational dimensionality and complexity while maintaining sufficient accuracy for evaluating the local contact pressure and oil-film thickness distributions. Based on this geometric simplification, further assumptions and boundary conditions were introduced to establish a complete contact–lubrication–stress coupling framework. The model considers both elastohydrodynamic lubrication (EHL) and boundary direct contact (BDR), and a load-sharing function is applied to ensure a smooth transition between the two regions. The pressure distribution within the contact area is solved from the EHL equations under the boundary conditions of pressure continuity at the inlet and zero pressure at the outlet.

In addition, the instantaneous contact stress is superimposed with the residual stress field of the gear surface. The residual stress varies with depth according to X-ray diffraction measurements, showing compressive stress near the surface that gradually decreases with depth. These assumptions and boundary settings ensure that the model accurately describes the interaction between contact pressure, residual stress, and surface roughness effects.

**Figure 6:**
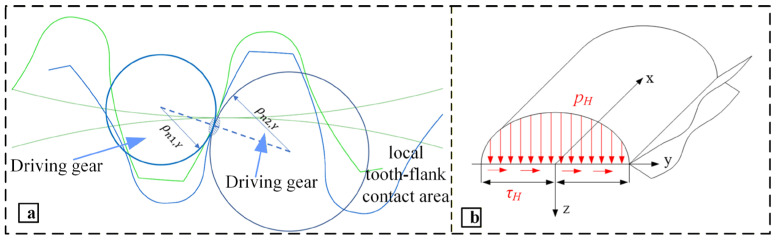
Schematic diagram of equivalent gear pair meshing and force distribution on the meshing surface. (**a**) Schematic diagram of gear pair meshing, (**b**) force state of the meshing surface.

The simplified model of gear meshing is shown in Figure 6a. The spatial coordinate system constructed at the meshing surface is shown in Figure 6b. The agreed meshing surface is x-axis along its axial direction, y-axis in the horizontal direction, and z-axis in the vertical direction. The spatial position and force status of the entire meshing surface have a transparent three-dimensional coordinate reference system and intuitively reflect the distribution and direction of contact stress and surface shear stress on the meshing surface in the simplified model.

Based on the contact theory of elasticity, the test gear pair’s surface and teeth body stress are calculated using the established gear surface stress model [20]. Liu [21] proposed a new method for calculating the stress field considering multiaxial loading conditions. This method can more accurately analyze the stress distribution in gears, which may provide new ideas for improving the gear stress field model in this study. The normal stresses in the three principal directions of the tooth surface (axial, tangential, and normal) are calculated using Equations (1)–(4), and the corresponding shear-stress components on the tooth surface are given as follows.

(1) $$\begin{array}{l} 1\sigma_{\;yy}=\left\{\begin{array}{l} -2\mu \left(\frac{y}{b_{h}}-\sqrt{{\left(\frac{y}{b_{h}}\right)}^{2}}-1\right)\cdot \frac{b_{h}}{\Delta },y\geq b_{h}\\ -\left(\sqrt{1-{\left(\frac{y}{b_{h}}\right)}^{2}}+2\mu \cdot \frac{y}{b_{h}}\right)\cdot \frac{b_{h}}{\Delta },-b_{h}\leq y\leq b_{h}\\ -2\mu \left(\frac{y}{b_{h}}+\sqrt{{\left(\frac{y}{b_{h}}\right)}^{2}}-1\right)\cdot \frac{b_{h}}{\Delta },y\leq -b_{h} \end{array}\right. \end{array}$$

(2) $$\sigma_{xx}=\left\{\begin{array}{l} -2v\mu \left(\frac{y}{b_{h}}-\sqrt{{\left(\frac{y}{b_{h}}\right)}^{2}}-1\right)\cdot \frac{b_{h}}{\Delta },y\geq b_{h}\\ -2v\left(\sqrt{1-{\left(\frac{y}{b_{h}}\right)}^{2}}+\mu \cdot \frac{y}{b_{h}}\right)\cdot \frac{b_{h}}{\Delta },-b_{h}\leq y\leq b_{h}\\ -2v\mu \left(\frac{y}{b_{h}}+\sqrt{{\left(\frac{y}{b_{h}}\right)}^{2}}-1\right)\cdot \frac{b_{h}}{\Delta },y\leq -b_{h} \end{array}\right.$$

(3) $$\sigma_{zz}=\left\{\begin{array}{l} 0\\ -\sqrt{1-{\left(\frac{y}{b_{h}}\right)}^{2}}\cdot \frac{b_{h}}{\Delta }\\ 0 \end{array}\right.\begin{array}{l} y\geq b_{h}\\ -b_{h}\leq y\leq b_{h}\\ y\leq -b_{h} \end{array}$$

(4) $$\tau_{yz}=\left\{\begin{array}{l} 0\\ -\mu \sqrt{1-{\left(\frac{y}{b_{h}}\right)}^{2}}\cdot \frac{b_{h}}{\Delta }\\ 0 \end{array}\right.\begin{array}{l} y\geq b_{h}\\ -b_{h}\leq y\leq b_{h}\\ y\leq -b_{h} \end{array}$$

## 3. Stress Distribution Analysis of Teeth Body

The regular stress calculation expressions of the three directions of the teeth body are shown as (5)–(7), and the shear-stress-calculation expressions of the teeth body are shown as (8).

(5) $$\sigma_{yy}=-\frac{b_{h}}{\pi \Delta }\left\{\begin{array}{l} z\left(\frac{b_{h}^{2}+2z^{2}+2y^{2}}{b_{h}}\phi_{1}-\frac{2\pi }{b_{h}}-3y\phi_{2}\right)+\\ \mu \left[\left(2y^{2}-2b_{h}^{2}-3z^{2}\right)\cdot \phi_{2}+\frac{2\pi y}{b_{h}}+2\left(b_{h}^{2}-x^{2}-z^{2}\right)\cdot \frac{y}{b_{h}}\phi_{1}\right] \end{array}\right\}$$

(6) $$\sigma_{xx}=-\frac{2vb_{h}}{\pi \Delta }\left\{\begin{array}{l} z\left(\frac{b_{h}^{2}+z^{2}+y^{2}}{b_{h}}\phi_{1}-\frac{\pi }{b_{h}}-2y\phi_{2}\right)+\\ \mu \left[\left(y^{2}-b_{h}^{2}-z^{2}\right)\cdot \phi_{2}+\frac{\pi y}{b_{h}}+\left(b_{h}^{2}-y^{2}-z^{2}\right)\cdot \frac{y}{b_{h}}\phi_{1}\right] \end{array}\right\}$$

(7) $$\sigma_{z}=-\frac{b_{h}}{\pi \Delta }\left[z\left(b_{h}\phi_{1}-y\phi_{2}\right)+\mu z^{2}\phi_{2}\right]$$

(8) $$\tau_{yz}=-\frac{b_{h}}{\pi \Delta }\left\{z^{2}\phi_{2}+\mu z^{2}\left[\left(x^{2}+2y^{2}+2z^{2}\right)\frac{z}{b_{h}}\phi_{1}-\frac{2\pi z}{b_{h}}-3yz\phi_{2}\right]\right\}$$

(9) $$\phi_{1}=\frac{\pi \left(M+N\right)}{M\cdot N\sqrt{2M\cdot N+2y^{2}+2z^{2}-2b^{2}}}$$

(10) $$\phi_{2}=\frac{\pi \left(M-N\right)}{M\cdot N\sqrt{2M\cdot N+2y^{2}+2z^{2}-2b^{2}}}$$

(11) $$M=\sqrt{{\left(b_{h}+y\right)}^{2}+z^{2}}$$

(12) $$N=\sqrt{{\left(b_{h}-y\right)}^{2}+z^{2}}$$

(13) $$\Delta =\frac{2}{\left(\frac{1}{\rho_{n1}}+\frac{1}{\rho_{n2}}\right)}\left(\frac{1-v_{1}^{2}}{E_{1}}+\frac{1-v_{2}^{2}}{E_{2}}\right)$$

where *z* is the distance from the teeth surface, mm; *E*_1_/*E*_2_ is driving/driven gear elastic modulus, Pa; *ρ*_n1_/*ρ*_n2_ is the equivalent curvature radius of drive/driven gear, mm; *b*_h_ is contact half-width, mm; *μ* is friction coefficient; and *ν* is Poisson’s ratio.

## 4. Instantaneous Contact Stress Calculation

The first stage of model construction accurately calculates the elastic stress distribution at each time point in the cycle. The model reflects the geometric characteristics of the contact interface, including the actual roughness of the gear teeth surface. When meshing, it uses mixed lubrication theory to accurately simulate the complex mechanical interaction between the driving and driven gear and accurately estimate the load distribution acting on the contact area. Specifically, a load-sharing mixed-lubrication scheme is used: the total load is partitioned between the EHL part and the boundary contact part by the weighting relations in Equations (20)–(25), while the force equilibrium is enforced by Equation (19), following the classical Hertz/EHL and mixed-lubrication formulations. Accuracy was evaluated by consistency and convergence checks: the model recovers the pure-EHL Hertzian solution for large film parameters and the boundary-contact limit for small λ, while the load balance is satisfied within ≤1% and mesh/time-step sensitivity remains below 3%. Stress calculations are performed at each meshing point in the model.

(14) $$F(t)=F^{\mathrm{EHD}}+F^{\mathrm{BDR}}$$

where *F* is the normal contact load per unit length, N; *F* ^EHD^ is complete elastic-hydrodynamic lubrication under load, N; and *F* ^BDR^ is part of the load borne by the direct contact of the teeth surface, N.

To describe the internal relationship between the above load and λ, the load divide function f_λ_ (λ) is used as follows.

(15) $$F^{\mathrm{EHD}}=f_{\lambda }(\lambda )\cdot F$$

(16) $$F^{\mathrm{BDR}}=(1-f_{\lambda }(\lambda ))\cdot F$$

Pressure is closely related to measurable or known parameters such as surface velocity, fluid viscosity, and density [22], and its expression equation is as follows.

(17) $$\frac{\partial }{\partial x}\left[\frac{\rho h^{3}}{\eta }\cdot \frac{\partial p}{\partial x}\right]=12u\frac{\partial (\rho h)}{\partial x}+12\frac{\partial (\rho h)}{\partial t}$$

where *ρ* is the lubricating oil density, kg/m^3^; *η* is the lubricating oil viscosity, Pas; *u* is the entrainment velocity, m/s; *p* is the oil-film pressure, Pa; and *h* is the oil-film thickness, m.

The lubricating oil-film thickness equation describes the liquid film thickness formed by the lubricating oil between two contact surfaces under lubrication, and the equation is as follows:

(18) $$h(x,t)=h_{0}(t)+\frac{x^{2}}{2R(t)}-\frac{2}{\pi E_{r}}\int_{-\infty }^{x}p(\zeta ,t)\ln{\left(x-\zeta \right)}^{2}d\zeta$$

where *h*_0_ is the central oil film thickness at the center of the contact ellipse; m, *R* is the equivalent radius of curvature of the meshing point, m; and E^*^ is the equivalent elasticity modulus of the two contact bodies, Pa.

The sum of the pressure carried in the line contact area equals the point load applied per unit length, so the load balance equation follows.

(19) $$W=W_{a}+\omega_{c}=\iint \left[p_{a}(x,t)+p_{c}(x,t)\right]dx$$

The contact stress distribution can be regarded as the result of continuous interpolation between the complete elastic-hydrodynamic lubrication problem and the boundary lubrication problem of the oil film. The import function *g*(*x*) [23] describes, at a specific location (*x*), the distribution proportion between the complete elastic-hydrodynamic lubrication stress of the oil film *p*^EHD^ and the boundary lubrication stress *p*^BDR^ that together form the mixed-film *p*^MIX^ lubricating stress. This function *g*(*x*) should satisfy the following conditions:

(20) $$p^{\mathrm{MIX}}(x)=g(x)\cdot p^{\mathrm{EHD}}(x)+(1-g(x))\cdot p^{\mathrm{BDR}}(x)$$

(21) 0≤g(x)≤1

(22) $$\underset{\lambda \to 0}{\lim}\;g(x)=0$$

(23) $$\underset{\lambda \to +\infty }{\lim}g(x)=1$$

(24) $$p^{\mathrm{EHD}}(x)=f_{\lambda }(\lambda )\cdot p^{\mathrm{EHD}}(x)$$

(25) $$p^{\mathrm{BDR}}(x)=(1-f_{\lambda }(\lambda ))\cdot p^{\mathrm{BDR}}(x)$$

(26) $$p^{\mathrm{MIX}}(x)=p^{\mathrm{EHD}}(x)+p^{\mathrm{BDR}}(x)$$

Its contact stress *p*^EHD^ can be calculated as follows for complete elastic-hydrodynamic lubrication.

(27) $$p^{\mathrm{EHD}}=p_{0}\sqrt{1-{\left(\frac{x}{b_{h}}\right)}^{2}}$$

where *p*_0_ is maximum Hertzian pressure, MPa; *b_h_* is Hertz contact half-width, mm. For boundary lubrication, the contact stress *p*^BDR^ can be calculated as follows.

(28) $$h(x)=h_{0}(x)-\frac{2}{\pi E_{r}}\int_{-\infty }^{+\infty }\ln\left|\frac{x-x^{\prime }}{L(x)}\right|p^{\mathrm{BDR}}(x)dx^{\prime }$$

(29) $$\int_{-\infty }^{+\infty }p^{\mathrm{BDR}}(x)dx=F(x)$$

(30) $$\forall x:h(x)\geq 0\wedge p^{\mathrm{BDR}}(x)\geq 0$$

where *h*(*x*) is the distance between two meshing teeth surfaces. *E_r_* is equivalent to the elastic modulus.

(31) $$\tau^{\mathrm{MIX}}(x)=\tau^{\mathrm{EHD}}(x)+\tau^{\mathrm{BDR}}(x)$$

(32) $$\dot{\gamma }=-\frac{\tau_{L}}{\eta }\ln\left(1-\frac{\tau }{\tau_{L}}\right)$$

where *τ*_L_ is the ultimate shear stress equivalent to the elastic modulus.

(33) $$\mu^{\mathrm{EHD}}=\frac{{\bar{\tau }}^{\mathrm{EHD}}\cdot 2b_{h}}{F^{\mathrm{EHD}}}$$

where $\bar{\tau }$ is the average shear stress in the lubricating oil.

(34) $$\mu^{\mathrm{EHD}}=\frac{2a\tau_{L}}{F^{\mathrm{EHD}}}\left[1-\exp\left(-\frac{\eta \dot{\gamma }}{\tau_{L}}\right)\right]$$

(35) $$\tau^{\mathrm{EHD}}(x)=\mu^{\mathrm{EHD}}\cdot p^{\mathrm{EHD}}(x)$$

## 5. Fatigue Pitting Judgment

In the second stage, the instantaneous contact stress obtained from the previous calculation is systematically superimposed with the inherent residual stress of each meshing point before the start of meshing. Then, the macroscopic stress at each meshing point can be calculated. Subsequently, the Dang Van multiaxial fatigue criterion determines the fatigue failure life. Note that the classical Dang Van [24] form may overestimate the influence of compressive (hydrostatic) stress on fatigue strength—particularly for rolling contact fatigue—so we also refer the reader to recent modification proposals [25,26]. Areitioaurtena [27] also used the Dang Van criterion to analyze the micro-pitting propagation behavior considering residual stress. Their research can provide a reference for setting more accurate judgment criteria in this study. The introduction of residual stress is shown as follows.

(36) $$p_{H}(x)=p^{\mathrm{MIX}}(x)+\sigma_{rs}(x)$$

where *σ*_rs_(*x*) is the residual stress on the teeth surface.

Although the present simulations emphasize compressive residual stresses, the model is equally capable of incorporating tensile residual stresses, which increase the local stress amplitude and reduce the fatigue resistance of the surface layer. In future work, the quantitative influence of tensile residual stresses on micro-pitting initiation and propagation will be further analyzed.

In the initial development stage of fatigue cracks, the crack propagation path generally follows the plane where the maximum shear strain lies. This is because the shear stress borne by the material within this plane is the largest, making it the most favorable area for crack initiation and propagation. Under the assumption of material property isotropy, this plane of maximum shear strain coincides with the plane of maximum shear stress. Therefore, the maximum shear stress becomes a parameter closely related to measuring the tendency of fatigue crack initiation in materials. The Dang Van multiaxial fatigue criterion can be expressed as follows.

(37) $$\tau_{\max}+\alpha_{DV}\cdot p_{H}\leq \beta_{DV}$$

where *τ*_max_ is maximum shear stress, MPa; *p*_H_ is hydrostatic stress, MPa; *α*_DV_ and *β*_DV_ are Material parameters, which can be obtained through reverse torsion and bending fatigue tests.

Dang Van also gives the relationship between *α*_DV_ and *β*_DV_ as follows.

(38) $$\alpha_{DV}=\frac{3\tau_{-1}}{\sigma_{-1}}-\frac{3}{2},\;\beta_{DV}=\tau_{-1}$$

where *τ*_−1_ is the reverse torsional fatigue limit, MPa; *σ*_−1_ is the Bending Fatigue Limit and MPa; *τ*_−1_/*σ*_−1_ is the relationship between torsional and bending fatigue limits for the specific material. For carburized gear steel 18CrNiMo *τ*_−1_/*σ*_−1_ = 0.577, and *σ*_−1_ are obtained from the bending fatigue test, *σ*_−1_ = 702 MPa.

Under rolling contact conditions, the classical Dang Van (DV) formulation is not conservative under compressive hydrostatic stress. Its hydrostatic term tends to overestimate the adverse effect of compression, thereby reducing the predicted fatigue strength.

Let *S*_DV_ be the safety threshold of micro pitting bearing capacity, as shown in Formulas (39) and (40). According to the judgment criterion, when *S*_DV_ ≤ 1, it indicates that the material point has not reached the conditions for micro-pitting initiation during the cyclic stage. Hence, the possibility of micropitting on the teeth’ surface is minimal.

(39) $$\beta_{DV\mathrm{act}}=\tau_{\max}+\alpha_{DV}\cdot p_{H}$$

(40) SDV=βD VactβDV

## 6. Result and Analysis

Instead of repeating detailed derivations, the discussion now focuses on how surface roughness and residual stress affect the stress field and S_DV_ indicator. For clarity, we use S_DV_ as the decision metric: S_DV_ > 1 indicates a safe condition, while S_DV_ ≤ 1 suggests possible crack initiation.

### 6.1. Oil Film Pressure and Oil Film Thickness Change

According to the EHL model, the distribution of oil film pressure and oil film thickness at meshing point A is calculated when the teeth surface roughness *R*_a_ 0 μm, *R*_a_ 0.05 μm, *R*_a_ 0.15 μm, and *R*_a_ 0.25 μm, respectively, as shown in Figure 6.

As shown in Figure 6a, when the teeth surface roughness *R*_a_ 0 μm, the oil film thickness curve is smooth in the contact area [−*b*_h_, *b*_h_], and the minimum oil film thickness is 0.22 μm. The corresponding oil film pressure curve is smooth, the maximum oil film pressure is 1.50 GPa, and the oil outlet position has a “neck shrinkage” phenomenon.

**Figure 7:**
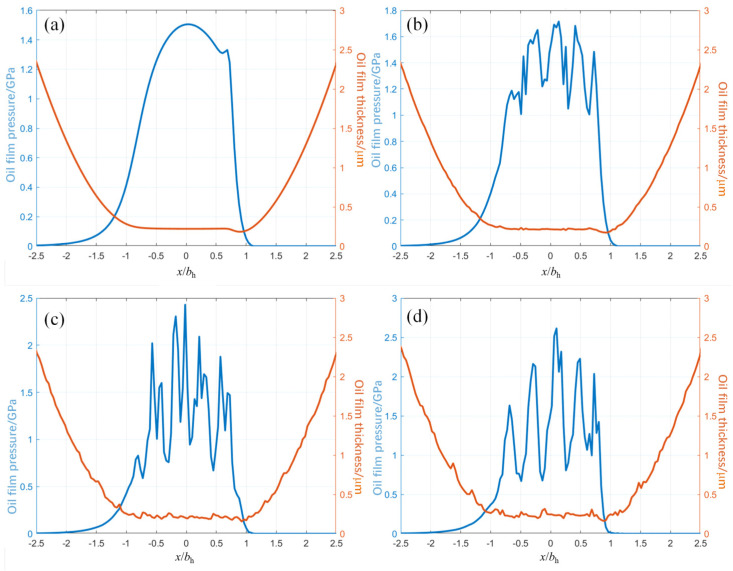
Distribution of teeth surface pressure (*P*) and oil film thickness (*h*) for different roughness levels. (**a**) *R*_a_ = 0 μm, (**b**) *R*_a_ = 0.05 μm, (**c**) *R*_a_ = 0.15 μm, and (**d**) *R*_a_ = 0.25 μm. *P* represents the oil-film contact pressure (left *y*-axis, GPa), and *h* denotes the local oil-film thickness (right *y*-axis, μm). The curves illustrate that increasing surface roughness.

As shown in Figure 7b, when the teeth surface roughness *R*_a_ 0.05 μm, the oil film thickness fluctuates slightly, and the corresponding oil film pressure curve fluctuates wildly. Meanwhile, the maximum oil film pressure reaches 1.68 GPa, an increase of 11.8%. As shown in Figure 7c, when the teeth surface roughness *R*_a_ 0.15 μm, the oil film thickness curve fluctuation continues to increase, and the corresponding oil film pressure curve fluctuation further increases. Moreover, the maximum oil film pressure reaches 2.43 GPa, an increase of 62.0%. As shown in Figure 7d, when the teeth surface roughness *R*_a_ 0.25 μm, the oil film thickness curve fluctuates violently, and the corresponding oil film pressure curve fluctuation is further intensified. Its maximum value reaches 2.61 GPa, an increase of 74.0%.

Using the minimum oil film thickness model and the elastic-hydrodynamic lubrication model, respectively, the calculated oil film thickness and oil film pressure distribution comparison curves are shown in Figure 7. On the one hand, as the roughness increases from 0 to 0.35 μm, the oil film thickness calculated by the minimum oil film thickness model decreases gradually, reducing by 72.7%; the oil film thickness calculated by the EHL model also decreases, but only decreases by 23.6%. On the other hand, with the increase in roughness, the oil film pressure calculated by the minimum oil film thickness model does not change. The model approximates the calculated oil film pressure, and the contact stress at the meshing point replaces the oil film pressure. On the contrary, the oil film pressure calculated by the EHL model increases by 70.0%.

In summary, in contrast to the minimum oil film thickness model, the EHL model considers the fluctuations in oil film pressure caused by the actual teeth’ surface roughness. This consideration provides accurate data support for the subsequent establishment of the micro-pitting bearing capacity model.

### 6.2. Distribution Change in Gear Teeth Shear Stress Field

**Figure 8:**
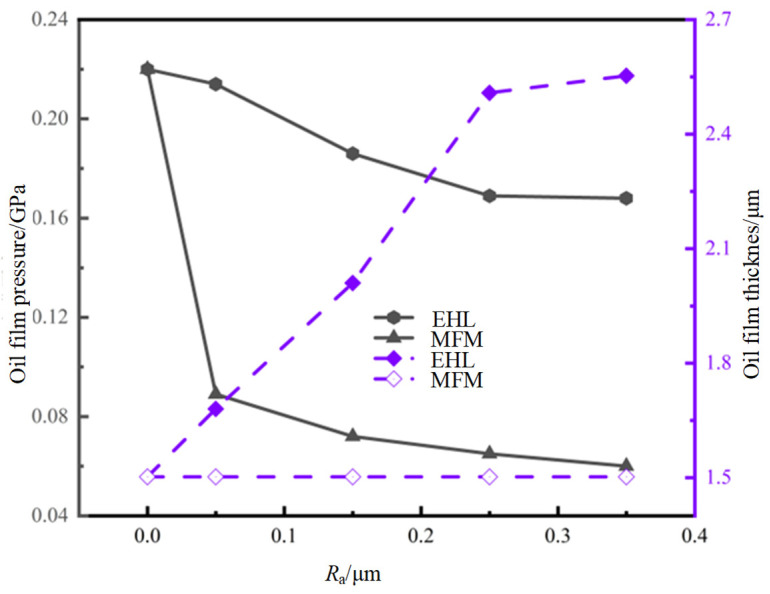
Variation curves of oil-film pressure and thickness for the two models.

**Figure 9:**
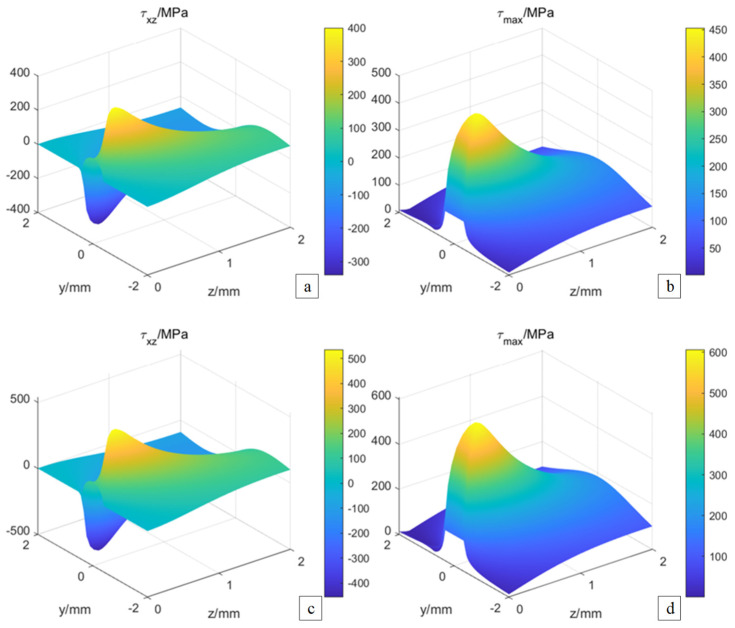
Shear stress and maximum shear stress of teeth surface. (**a**) shear stress of *R*_a_ 0 μm, (**b**) maximum shear stress of *R*_a_ 0 μm, (**c**) shear stress of *R*_a_ 0.25 μm, (**d**) maximum shear stress of *R*_a_ 0.25 μm.

Based on the established gear stress field model, the distribution of shear and maximum shear stress are calculated. As shown in Figure 8, when the teeth surface roughness *R*_a_ 0 μm, the shear stress distribution and the maximum shear stress distribution at the meshing point are calculated. Here, the shear stress distribution refers to the spatial variation in τ (x, z) within the tooth-surface layer, whereas the maximum shear-stress distribution represents the envelope of the peak values τₘₐₓ(z) at different subsurface depths. As shown in Figure 9a, the shear stress on the teeth surface initially increases from 0 to its maximum value in the counter-clockwise direction and then decreases back to 0. Subsequently, when the direction changes clockwise, it rises from 0 to the maximum again and then drops to 0. The maximum value of the shear stress is 74.6 MPa. As shown in Figure 9b, the maximum shear stress at the teeth surface shows a trend of increasing from 0 to the maximum and then decreasing to 0 in the y direction, and the maximum value is 74.8 MPa.

When the teeth surface roughness *R*_a_ 0.25 μm, the distribution of the shear stress and the maximum shear stress at the meshing point is shown in Figure 9c,d. The change trends of the shear stress and the maximum shear stress change area on the rough teeth surface are consistent with those on the smooth teeth surface. However, the maximum values differ. The maximum shear stress on the rough teeth surface is 124.5 MPa, representing a 66.89% increase compared to the smooth teeth surface. Additionally, another measurement shows that the maximum shear stress on the rough teeth surface is 124.9 MPa, a 66.98% increase relative to the smooth teeth surface.

Under different roughness, the shear stress and the maximum shear stress at the teeth surface change in the z-direction, as shown in Figure 9a,b. In the z direction, the shear stress increases and decreases. The position of the maximum shear stress is 0.24 mm from the surface depth. Moreover, the shear stress at the teeth surface gradually rises as the roughness increases. Regarding the maximum shear stress, the z-direction increases and decreases first, and its maximum position is 0.30 mm from the surface depth. Additionally, with the increase in roughness, the maximum shear stress at the teeth surface gradually increases. Based on the above analysis, the variation in teeth surface roughness affects the peak values of shear stress and maximum shear stress.

### 6.3. Effect of Teeth Surface Roughness on Fatigue Pitting

**Figure 10:**
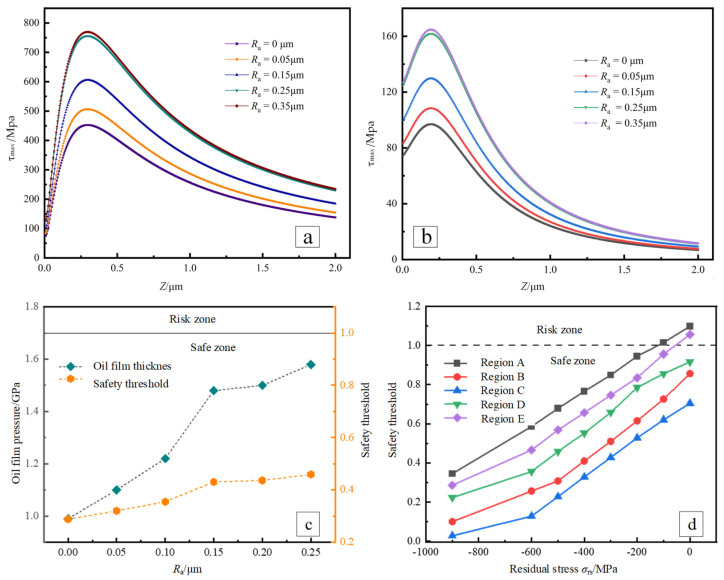
Shear stress, maximum shear stress, oil film pressure, and safety threshold distribution of different teeth surface roughness and residual stress. (**a**) Shear-stress distribution of tooth surface at different roughness levels; (**b**) Maximum shear-stress distribution along the depth direction; (**c**) Oil-film pressure and safety-threshold variation with roughness; (**d**) Safety-threshold distribution at five meshing points.

As shown in Figure 10c, the oil film pressure gradually increases with teeth surface roughness. When the teeth surface roughness *R*_a_ is less than 0.15 μm, the average growth rate of oil film pressure is 16.7%, and the growth rate of safety threshold is 13.3%; when the teeth surface roughness *R*_a_ is more significant than 0.15 μm, the oil film pressure growth slows down, and its average growth rate is only 2.67%. The growth rate of the safety threshold is 1.25%. The changing trend of the safety threshold is consistent with the changing trend of oil film pressure. When the teeth’ surface roughness Ra is less than 0.15 μm, the safety threshold increases rapidly; when the teeth’ surface roughness *R*_a_ is more significant than 0.15 μm, the growth of the safety threshold slows down. Therefore, increasing teeth surface roughness will decrease the bearing capacity of micro-pitting corrosion.

### 6.4. Effect of Teeth Surface Residual Stress on the Fatigue Pitting

According to the constructed micro-pitting carrying capacity model, the safety thresholds of micro-pitting at five main meshing points are calculated under different residual stresses, as shown in Figure 10d. With the decrease in residual compressive stress on the teeth surface, the safety thresholds at each meshing point increase linearly. The safety threshold at meshing point A is more significant than the other four points. It is proved that the risk of micro-pitting at meshing point A is more significant.

As shown in Figure 10d, taking meshing point A as an example, when the residual compressive stress of the teeth surface is 0, the safety threshold of micro-pitting corrosion is 1.098, and the risk of micro-pitting is greater. When the residual compressive stress increases to −100 MPa, the safety threshold decreases to 1.015, and the risk of micro-pitting decreases. When the residual compressive stress increases to −900 MPa, the safety threshold decreases by 68.6%, and the risk of micro-pitting further decreases. The analytical results were validated against measured residual-stress distributions and fatigue tests, showing consistent micro-pitting initiation trends.

With node C as the boundary, the safety threshold gradually decreases under the same residual stress level in the region below the pitch circle, and the safety threshold gradually increases under the same residual stress level in the meshing process from the meshing point C→D→E. This shows that the micro-pitting carrying capacity at node C is the largest, and the risk of micro-pitting at meshing points A and E. However, the safety threshold at meshing point A is more significant than at meshing point E. The safety threshold at meshing point B is more significant than that at meshing point D. The risk of micro-pitting in the meshing area is greater than the risk of micro-pitting in the meshing area. The risk of micro-pitting at meshing point A is the largest.

From a practical perspective, when the surface roughness exceeds Ra 0.6 μm, the increased contact and shear stress drive the S_DV_ value below 1, implying a high micro-pitting risk. In contrast, maintaining a compressive residual stress between −350 and −450 MPa ensures S_DV_ > 1, effectively enhancing surface fatigue resistance and extending gear service life.

A modified S_DV_-based Dang Van criterion was employed to mitigate the overestimation of compressive stress influence, calibrated using experimentally measured residual-stress data.

### 6.5. Fatigue Pitting Prediction Model Determination

**Figure 11:**
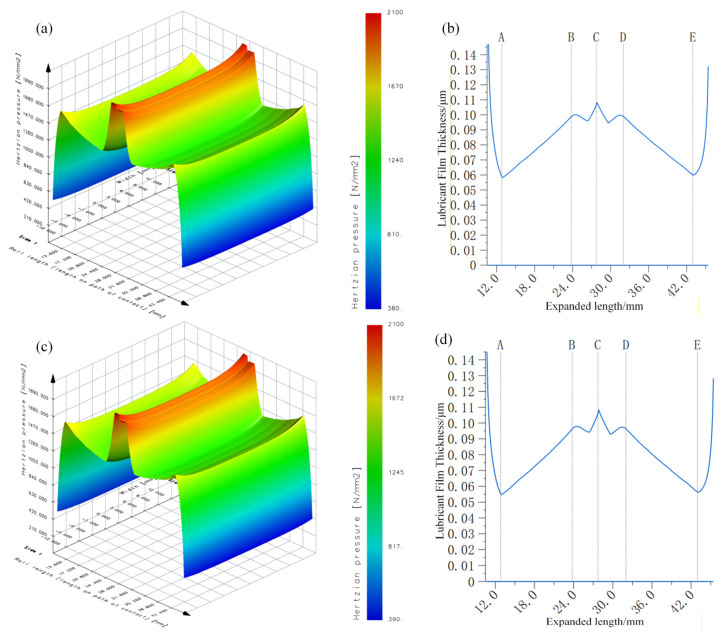
Contact stress and oil film thickness distributions based on the traditional model (**a**) contact stress of *R*_a_ 0.35 μm and (**b**) *R*_a_ 0.65 μm, (**c**) oil film thickness of *R*_a_ 0.35 μm and (**d**) *R*_a_ 0.65 μm.

Based on the minimum oil film thickness micro-pitting model, the gear micro-pitting model is proposed, where the oil film thickness, contact stress value, and load capacity determination value at the meshing point A of gears with two different roughness are calculated. Figure 11 represents the macro stress distribution obtained after coupling the contact load with the residual-stress field. Expanded length refers to the extended meshing width used for stress-distribution post-processing. Figure 11a,b illustrates the Hertz contact stress distribution of the teeth surface *R*_a_ 0.35 μm and *R*_a_ 0.65 μm. The contact stress on the tooth surface varies continuously along the meshing direction with a stress value of 1549.0 MPa at meshing point A, while the contact stress near the pitch line reaches its peak of 1912 MPa. When comparing the contact stress of gears of tooth surface roughness *R*_a_ 0.35 μm and *R*_a_ 0.65 μm, it is observed that the stress values are identical at each meshing point. Therefore, the micro-dot minimum oil film thickness model exhibits certain limitations due to its failure to consider the impact of tooth surface roughness on contact stress.

Figure 7c,d shows the oil film thickness distribution on the teeth surface, the teeth surface *R*_a_ 0.35 μm and *R*_a_ 0.65 μm. Along the meshing direction, the variation trend of oil film thickness is consistent with the variation trend of contact stress. When the tooth surface roughness is *R*_a_ 0.35 μm, the oil film thickness at the meshing point A is the smallest of 0.058 μm, and the oil film thickness at the node is the largest of 0.109 μm. When the roughness is *R*_a_ 0.65 μm, the oil film thickness at the meshing point A is 0.055 μm, which is reduced by 5.2%, and the oil film thickness at the node line is 0.109 μm, which has no significant change. Therefore, this indicates that the micro-dot minimum oil film thickness model has some limitations because it does not reflect the effect of tooth roughness on oil film thickness.

(41) $$K_{S}=\frac{1}{S_{DV}}$$

The safety coefficient (*K*ₛ) was obtained as *K*ₛ = 1/S_DV_ based on the Dang Van criterion; *K*ₛ > 1 indicates safety, while *K*ₛ ≤ 1 suggests potential micro-pitting initiation.

**Figure 12:**
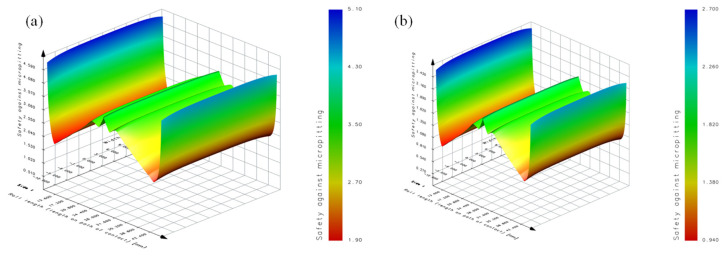
Distribution of micro-pitting safety coefficients based on the proposed model. (**a**) *R*_a_ 0.35 μm and (**b**) *R*_a_ 0.65 μm.

Figure 12 shows the distribution of the micro-pitting safety coefficient under different roughnesses. From Figure 12a, it can be seen that the trend of the micro-pitting safety coefficient along the engagement direction is consistent with that of the oil film thickness. When the roughness is *R*_a_ 0.35 μm, the micro-pitting safety coefficient at the engagement point A is the smallest of *S_λ_* 1.90, while the micro-pitting safety coefficient at the nodal line is the largest of *S_λ_* 3.80. From Figure 12b, it can be seen that the micro-pitting safety coefficient at the engagement point A is *S_λ_* 0.94 when the roughness is *R*_a_ 0.65 μm.

When the tooth surface roughness is *R_a_* = 0.35 μm, as shown in Table 4, based on the improved micro-pitting model proposed in this paper, the safety threshold is *S*_DV_ = 1.402S. Therefore, it can be determined that micro-pitting may occur on the tooth surface, which is consistent with the experimental results. However, according to the micro-pitting model based on the minimum oil-film thickness, the safety factor is *S*_λ_ = 1.90, indicating that micro-pitting would not occur on the tooth surface, which is inconsistent with the experimental results. It can thus be concluded that, under low surface roughness conditions, the improved micro-pitting model proposed in this paper can more accurately evaluate the load-carrying capacity of tooth-surface micro-pitting.

When the tooth surface roughness is *R*_a_ = 0.65 μm, as shown in Table 5, based on the improved micro-pitting model proposed in this paper, the safety threshold is *S*_DV_ = 2.779. Therefore, it can be determined that micro-pitting may occur on the tooth surface, which is consistent with the experimental results. According to the micro-pitting model based on the minimum oil-film thickness, the safety factor is S_λ_ = 0.94, indicating that micro-pitting may also occur on the tooth surface, which is consistent with the experimental results. It can thus be concluded that, under high surface roughness conditions, both micro-pitting models can accurately determine the load-carrying capacity of tooth-surface micro-pitting.

When the tooth surface roughness is *R*_a_ 0.65 μm, the proposed model has a safety threshold of *S*_DV_ 2.779, while the micro-pitting model of the minimum oil film thickness has a safety threshold of *S*_DV_ 0.94. The experimental observations confirmed that micro-pitting occurs on the tooth surface, consistent with the simulation results. The micro-pitting models all accurately determine the bearing capacity of teeth surface micro-pitting under high roughness. Here, “high roughness” refers to *R*_a_ ≥ 0.55 µm, where asperity contacts exceed 10% and EHL film continuity partly breaks down. The model accuracy was validated experimentally, showing a prediction error within ±10%. residual stress in the teeth surface stress field When the tooth surface roughness is *R*_a_ 0.35 μm, the proposed model has a safety threshold *S*_DV_ 1.402, and it determines that the micro-pitting occurs on the teeth surface, which is consistent with the test results. The micro-pitting model of the minimum oil film thickness has a safety threshold of *S*_DV_ 1.90, and it determines that micro-pitting does not occur on the teeth surface, which is inconsistent with the test results. The proposed model under lower roughness is more accurate in determining the micro-pitting bearing capacity of the teeth surface.

The results of the gear fatigue tests are shown in Table 6, and the data reveal a close relationship between the micro-pitting failure life of gears and the surface roughness. When *R*_a_ = 0.35 μm, micro-pitting initiates after 6 × 10^6^ cycles of operation, and the failure criterion is reached after 1.0 × 10^7^ cycles. When the tooth surface roughness increases to *R*_a_ = 0.45 μm, compared with the gear with *R*_a_ = 0.35 μm, the initiation of micro-pitting occurs 2.6 × 10^6^ cycles earlier, and the failure life is reduced by 4.0 × 10^6^ cycles. When the tooth surface roughness further increases to *R*_a_ = 0.55 μm, compared with the gear with Ra = 0.45 μm, the formation of micro-pitting is advanced by an additional 1.4 × 10^6^ cycles, and the failure life is reduced by 1.6 × 10^6^ cycles. When the tooth surface roughness increases to *R*_a_ = 0.65 μm, compared with the gear with *R*_a_ = 0.55 μm, the initiation of micro-pitting occurs 1.0 × 10^6^ cycles earlier, and the failure life is shortened by 1.0 × 10^6^ cycles.

## 7. Conclusions

In this paper, a fatigue pitting prediction model of gear steel considering teeth roughness and residual stress is proposed, and an analytical study of factors affecting micro-pitting load-bearing capacity is carried out. It should be noted that the proposed model assumes homogeneous material properties and simplified lubrication conditions, which may differ from the actual gear contact environment. Future work will focus on incorporating material heterogeneity and more realistic elastohydrodynamic lubrication behavior to improve the model’s accuracy. The results are shown as follows.

(1) When the tooth surface roughness is higher, the micro-pitting models accurately determine the bearing capacity of teeth surface micro-pitting under high roughness. However, when the tooth surface roughness is more minor, the proposed model under lower roughness is more accurate in determining the micro-pitting bearing capacity of the tooth surface.
(2) Based on the elastic-hydrodynamic lubrication model considering the tooth surface roughness, the larger the mean value of tooth surface roughness is, the more pronounced the fluctuation of oil film pressure on the tooth surface. The increment of tooth roughness from *R*_a_ 0 μm to *R*_a_ 0.25 μm caused the oil film pressure to increase by 74.0% and the micro-pitting safety threshold to increase by 50.0%.
(3) Based on the elastic-hydrodynamic lubrication model considering residual stress. When the residual stress on the tooth surface increases from 0 MPa to −900 MPa, the corresponding micro-pitting safety threshold is reduced by 68.6%, and the risk of micro-pitting emergence is significantly reduced. During the whole meshing process, the risk of micro pitting at the engagement point is greater than that of micro pitting at the disengagement point.

The proposed model can also be applied in practical engineering to assess and improve the micro-pitting resistance of gears used in high-load transmission systems, such as wind-turbine gearboxes, vehicle transmissions, and railway traction drives. Integrating the S_DV_-based evaluation approach into design and maintenance processes enables optimization of surface-treatment parameters and residual-stress control, thereby enhancing fatigue durability and preventing early micro-pitting failures.

Future work will focus on extending the proposed model to different gear geometries, materials, and loading conditions. In addition, broader experimental validation and integration with multi-scale fatigue models will be conducted to further enhance the model’s generality and predictive accuracy.

## Figures and Tables

**Table 1:** Geometrical parameters of the test gear.

Parameter	Symbol	Pinion/Driving Gear	Wheel/Driven Gear
Center distance (mm)	*a*	160	160
Number of teeth	*z*	24	25
Module (mm)	*m*	6.5	6.5
Pressure angle (°)	*α*	20	20
Helix angle (°)	*β*	0	0
Face width (mm)	*b*	34	30
Addendum modification coefficient	*x*	0.0735	0.0439
Addendum circle diameter (mm)	*d_a_*	169.31	175.42
Pitch circle diameter (mm)	*d*_0_	156	162.5
Accuracy grade	*/*	GB/T 10095.1–2022 [17]	GB/T 10095.1–2022 [17]
Yield limit (MPa)	*σ* *_−_*_1_	702	702
Fatigue limit (MPa)	*τ* *_−_*_1_	405	405

**Table 2:** Chemical composition of the gear material (18CrNiMo7-6).

Element	Specification (Wt%)	Measured Value (Wt%)	Element	Specification (Wt%)	Measured Value (Wt%)
C	0.17–0.21	0.190	Cr	1.60–1.80	1.680
Si	≤0.40	0.270	Ni	1.50–1.70	1.520
Mn	0.60–0.90	0.740	Mo	0.25–0.35	0.300
P	≤0.010	0.008	Cu	<0.20	0.070
S	≤0.010	0.009	Al	0.02–0.04	0.022

**Table 3:** Lubrication parameters for the test.

Parameter	Value
Lubrication method	Spray lubrication
Lubricant	L-CKD 68
Pressure–viscosity coefficient (m^2^/N)	0.0184
Kinematic viscosity ν_40_ (mm^2^/s)	68
Kinematic viscosity ν_100_ (mm^2^/s)	8.8
Lubricant density ρ_15_ (kg/m^3^)	880

**Table 4:** Comparison of micro-pitting prediction results from two models at surface roughness Ra = 0.35 μm.

Comparison Model	Tooth Surface Stress/MPa	Safety Factor/Value	Predicted Micro-Pitting Bearing Capacity Result
Oil-film thickness model	1549.0	*S*λ = 1.90	No micro-pitting predicted
Present improved model	2830.0	*S_DV_ =* 1.402	Micro-pitting predicted

**Table 5:** Comparison of micro-pitting prediction results from two models at surface roughness Ra = 0.65 μm.

Comparison Model	Tooth Surface Stress/MPa	Safety Factor/Value	Predicted Micro-Pitting Bearing Capacity Result
Oil-film thickness model	1549.0	*S*λ = 0.94	Micro-pitting predicted
Present improved model	3500.0	*S*_DV_*=* 2.779	Micro-pitting predicted

**Table 6:** Micro-pitting test results of polished gears.

Surface Roughness *R*a/μm	Test Result	Micro-Pitting Initiation/×10^4^ Cycles	Micro-Pitting Failure/×10^4^ Cycles
0.35	Micro-pitting observed	600	1000
0.45	Micro-pitting observed	340	960
0.55	Micro-pitting observed	200	800
0.65	Micro-pitting observed	100	700

## Data Availability

The original contributions presented in this study are included in the article. Further inquiries can be directed to the corresponding author.
